# Supervised on-site dosing in injectable opioid agonist treatment-considering the patient perspective. Findings from a cross-sectional interview study in two German cities

**DOI:** 10.1186/s12954-023-00896-6

**Published:** 2023-11-01

**Authors:** Zoe Friedmann, Hans-Tilmann Kinkel, Claudia Kühner, Andreas Zsolnai, Inge Mick, Annette Binder

**Affiliations:** 1 Charité Universitätsmedizin Berlin (Medical University Hospital Charité Berlin), Charitéplatz 1, 10117 Berlin, Germany; 2 Praxiskombinat Neubau, Schwerpunktpraxis für Suchtmedizin (Outpatient Clinic for Addiction Medicine), Ruschestraße 103, 10365 Berlin, Germany; 3 Schwerpunktpraxis für Suchtmedizin Stuttgart (Outpatient Clinic for Addiction Medicine), Kriegsbergstraße 40, 70174 Stuttgart, Germany; 4 Department of Psychiatry and Psychotherapy, Charité Universitätsmedizin Berlin (Medical University Hospital Charité Berlin), Charitéplatz 1, 10117 Berlin, Germany; 5 Department of General Psychiatry and Psychotherapy, Addiction Medicine and Addiction Research Section, University Hospital Tuebingen, University of Tuebingen, Calwerstraße 14, 72076 Tuebingen, Germany; 6 DZPG (German Centre for Mental Health), Tuebingen, Germany

**Keywords:** Injectable opioid agonist treatment, Opioid use disorder, Take-home doses, Dosing policies, Patient perspective, Qualitative research

## Abstract

**Background:**

Injectable opioid agonist treatment (iOAT) is an effective option to support people living with opioid use disorder (OUD) who have not sufficiently benefitted from oral OAT. However, iOAT has been criticised based on theoretical and practical grounds for its dosing policies: Current regulations demand supervised, on-site application and require patients to frequently visit their treatment facility. The current study aims to investigate how patients experience on-site application and derive strategies to enhance the acceptability and effectiveness of iOAT-delivery.

**Methods:**

This article is based on semi-structured interviews with 27 individuals currently or previously in iOAT in two German outpatient iOAT-clinics. We undertook an inductive qualitative content analysis, which included blinded, independent coding and the analysis of individual cases.

**Results:**

Comments regarding on-site application and daily visits to the clinic were grouped into *positive and negative aspects*, *iOAT as the best alternative option, facilitators of daily visits*, and *suggestions for improvement*. Positive aspects took the factors stability and social support in regard. Negative aspects ranged from general inconveniences to major impediments to individuals' daily lives and towards achieving psychosocial goals. Participants reported rigorous adherence to iOAT's treatment regime, often due to a perceived lack of alternative options. Meeting iOAT's demands was eased by the patients’ coping-strategies and through facilitating measures implemented by iOAT-clinics. Despite acknowledgement of the potential detriments from easing regulations, take-home arrangements were frequently suggested by participants to improve iOAT.

**Conclusions:**

Being required to attend the clinic for supervised iOAT-application is not experienced uniformly. While clinics can support their patients to cope with strict regulations, alternative approaches to iOAT-application should be considered to accommodate patients' individual needs. Examples from other treatment modalities (e.g., remote supervision and delivery services) might aid to reconcile individualisation while providing adequate safety measures and improve iOAT in the long term.

**Supplementary Information:**

The online version contains supplementary material available at 10.1186/s12954-023-00896-6.

## Background

Injectable opioid agonist treatment (iOAT) with diamorphine (DAM) or hydromorphone is an effective approach to support individuals living with severe opioid use disorder (OUD) who have not sufficiently benefitted from oral opioid agonist treatment (oOAT) [1]. People previously considered unresponsive to treatment have been shown to improve their physical and mental health, social integration, and treatment retention in iOAT [2, 3]. DAM-based iOAT has been available in Germany since 2009 [4]. Despite its proven effectiveness, the implementation of iOAT remains worryingly limited in Germany [5, 6].

In iOAT, patients inject their substitute in specialised outpatient clinics under the supervision of healthcare staff. While in oOAT patients are often granted take-home doses or “carries” of their substitute [7], this is usually not available to those in iOAT [8]. Notably, some countries eased their regulations in response to the COVID-19 pandemic [9], whereas Germany continues to unvaryingly require supervised, on-site application of injectable DAM. To receive their medication, patients in iOAT must therefore attend their treatment facility, often multiple times a day as diamorphine is relatively short-acting [10, 11]. From a public health point of view, supervised application in OAT is implemented due to concerns about diversion and misuse, which can cause extensive individual and societal harm [12, 13]. In iOAT, this is enhanced by the higher propensity of adverse events including overdoses resulting from the substances used and the intravenous nature of the application [14, 15]. Nevertheless, the inflexible requirement for supervised, on-site application in iOAT has been a source of criticism [16, 17] as it likely impedes treatment retention [18, 19] and therefore merits further investigation.

### The effect of on-site supervised application in OAT

Past empirical research on supervised OAT and its obligatory clinical setting has had mixed results [20, 21]. On the one hand, on-site application increases the “treatment burden” associated with OAT (e.g., through time and money spent commuting) [22] and can interfere with patients' social life, formal employment, and leisure activities [2, 23–25]. Furthermore, strict dosing policies have been described as a means of exerting social control [26–28] and can contribute to the stigmatisation of people receiving OAT [29–32]. On the other hand, daily visits can provide stability and regular access to medical assistance [33], fill “the void” left by ceasing to use street drugs [34], and contribute to both the patients' “re-socialisation” [35] and the formation of a supportive community within OAT-clinics [36]. Additionally, the regular contact with clinic staff might enhance the “therapeutic alliance” [37] and positively contribute to the psychosocial components of iOAT [19, 38], particularly when staff have an accepting approach to supervised injecting [39].

Considering these diverging effects, it comes as little surprise that the acceptability of supervised, on-site OAT is context-dependent and highly variable among patients in both oOAT [40] and iOAT [33, 38, 41]. Generally, daily attendance is less challenging when OAT is provided in a non-stigmatising setting which is conveniently located (e.g., close to patients' home or workplace), has extended service hours, and provides efficient services [42, 43]. In oOAT, short-term supervision with the prospect of take-home doses greatly enhances the acceptability of strict policies among individuals in treatment [44, 45]. This is crucial, as “singling out iOAT from the option of carries” [17] likely amplifies the importance of supervised application in iOAT.

While past research has identified the requisite daily visits as a critical issue among many individuals in iOAT, there is a lack of explicit investigation into how patients engaged in iOAT experience on-site application. In a different manuscript submitted for publication elsewhere [46], we report on the negative influence of daily visits on treatment-initiation among individuals currently not engaged in iOAT. In the current article, we focus on the significance of the supervised, on-site application in iOAT for individuals in treatment. Our aim is to investigate how patients experience on-site application and consequently to derive strategies to enhance the acceptability and effectiveness of iOAT-delivery both within and beyond Germany.

## Methods

The data for this article stem from a qualitative study based on semi-structured interviews with 34 people (a) currently in, (b) previously in, or (c) eligible for but never having been in iOAT. The study aimed to explore the patient perspective on iOAT, particularly regarding ways to improve the therapy and its acceptability among people living with OUD. All individuals meeting German eligibility criteria for iOAT (minimum age 23 years; opioid use for at least five years; ongoing intravenous use; two previous attempts in OUD-treatment, one of which being no less than 6 months in oOAT; and serious health impairments due to continued drug use [4]) and who were able to provide informed consent were eligible to participate in the study. To explore the real-life significance of daily visits in iOAT, in the current article we focus on the 27 participants who were (a) currently or (b) previously in iOAT.

### Data gathering

Based on the study’s objective and on prior research in implementation science, we developed separate semi-structured interview guides for participants currently or previously in iOAT (see Additional file 1). The guides shared similarities in their aim to gain a rich understanding of experiences regarding iOAT and included open questions about the initiation and discontinuation of iOAT, experiences in treatment, and ways to improve the therapy. Along with the study's objectives, we discussed the interview guides in a focus group including both individuals who live with OUD and professionals who provide psychosocial support in iOAT-clinics. During the focus group session, it was confirmed that our work was acceptable, feasible, and of relevance for people living with OUD [47].

Guided by the concept of “information power” from Malterud et al. [48] and by practical considerations, we agreed to include an initial number of participants of 16 individuals currently partaking in iOAT and four individuals currently partaking in oOAT after having discontinued iOAT. Following approval from the ethics committee of Landesärztekammer Baden-Württemberg (AZ: F-2022-002), we began recruitment in April of 2022. We recruited participants from two outpatient iOAT-clinics in Berlin and Stuttgart. Both clinics provide injectable and oral OAT, psychosocial support, and general medical services to several hundreds of patients. First, the clinics prepared a list of patients eligible for the study. Stratified to reflect the gender and age of the patient population at each site, individuals were then randomly selected from these lists. Clinic staff approached the selected individuals and provided them with study information. It was stressed that participation was entirely voluntary and the decision to take part was independent of the participants' care. Upon interest to participate, an informed consent form was completed and interviews were scheduled at a time convenient for participants.

Supported by preliminary analyses and throughout the data gathering process, the adequacy of the initially set number of participants was continuously evaluated. In this process, we aimed for a deep and nuanced understanding of participants' experiences and continued scheduling interviews until no new topics emerged. In general, participants appeared to welcome the opportunity to share their experiences and all individuals we approached agreed to participate. Four individuals who had given consent could not be included in the study as they repeatedly missed their scheduled interviews. Ultimately, 23 participants who were undergoing iOAT and four participants undergoing oOAT who had discontinued iOAT were included.

ZF, a female medical student unknown to the participants and not professionally active in OUD-care, conducted all interviews between May and August of 2022 in private rooms in the respective clinics. Upon written and oral informed consent, ZF audio-recorded the interviews (ranging in length from 18 to 60 min) and maintained reflexive field notes. Through open questions, ZF invited participants to lead the course of the interview and set priorities for themselves. Thus, the aspects presented in the results section of this article arose spontaneously from the participants themselves, albeit to varying degrees. Following the interviews, participants were encouraged to contact ZF for any further inquiries or to obtain transcripts of their interviews. All participants, including those consulted prior to data collection and publication, received a compensation of 20€ in cash, which was self-financed by the research team. Measures were taken to mitigate potential conflicts of interest and biases arising from this arrangement (e.g., repeated critical reflections and consulting Charité's *Office for Research Integrity*).

### Data analysis

Parallel to data gathering we began analysing data and oriented our approach according to Tong et al.'s [49] *consolidated criteria for reporting qualitative studies*. To produce a thorough audit trail, ZF kept a log of all analytical reflections and methodological decisions made during the research process. Following software-assisted (NVivo 2022) manual transcription by ZF, de-identified transcripts were assigned gender-appropriate pseudonyms and imported into MaxQDA software (2022) for qualitative content analysis [50]. We followed an inductive approach, which started with a thorough and iterative examination of all interview transcripts to identify initial patterns. ZF, who led the data analysis, then inductively derived major categories and increasingly differentiated subcategories from the data. Supported by reflexive memo-writing, we developed a codebook that contained a comprehensive list of our categories and their definitions. The codebook served as a detailed reference guide to ensure that coding decisions were consistent and transparent. The emerging category system was elaborated during blinded, independent coding by PT. Here, discrepancies and difficulties in coding were discussed and adaptions to the category system were made until a consensus was reached. In total, about 20% of the material was coded independently. To identify variations in participants' accounts and to enhance the robustness of our analysis, cross-case analyses were repeatedly compared with individual interview transcripts.

As our understanding of the data evolved, we continuously refined our analytical approaches. In regular verification meetings, we discussed the category system and how to interpret emerging patterns in the data. During the verification meetings, researchers involved in participants' care only reviewed de-identified illustrative quotes and we aimed to critically reflect upon our respective roles, responsibilities, and potential biases. Additional intersubjective validation was sought by presenting the research in all stages at interdisciplinary colloquia and interpretation groups. Furthermore, we individually consulted three people living with OUD prior to the publication of this article in order to ensure that our interpretations aligned with informed lived experiences. As the feedback received during these consultations was affirmatory, it did not result in significant changes to the manuscript.

## Results

Of the 27 participants who had ever been in iOAT, 20 were cisgender male and seven cisgender female. Participants were aged 31–59 (mean 43) years and included people of colour and non-German citizens. For many participants, iOAT was interwoven with phases of oOAT, complete abstinence, or the consumption of street drugs. Participants' approximated experiences of continuous iOAT ranged in length between two weeks and nine years (mean approximately 2.5 years). In order to protect participants' identity, further information on diversity metrics is not disclosed.

**Fig. 1:**
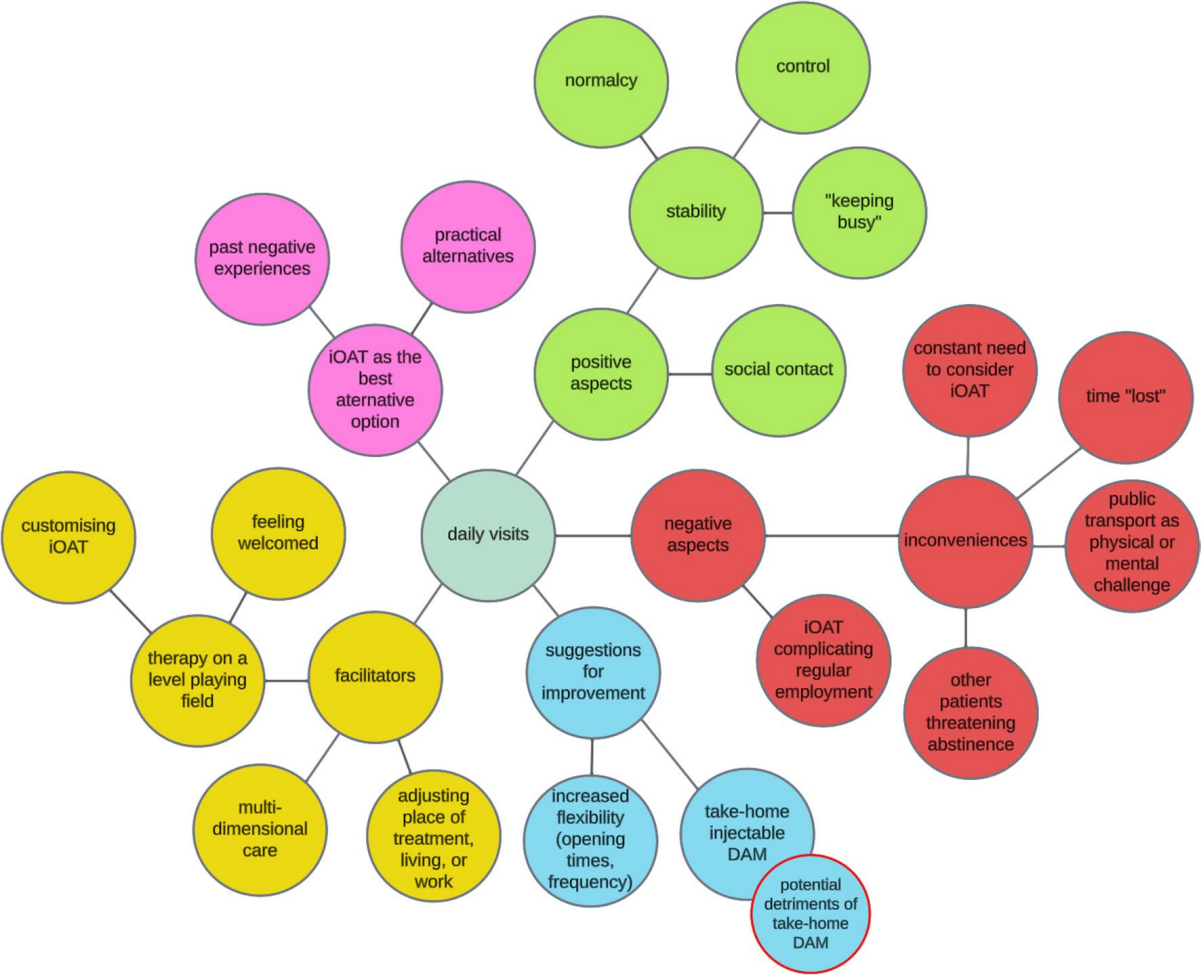
The major category “daily visits” was used to guide the current article and is shown with its subcategories

Our analysis yielded several major categories, e.g., “the intravenous application in iOAT” and “DAM as a substance”. The current article focuses on the major category of “daily visits to the clinic”. In reporting our results, we follow the subcategories of the major category on daily visits as shown in Fig. 1. The illustrative quotations presented below were translated from German using the forward–backward translation technique to ensure concurrent validity and alignment with the original data.

### Positive aspects

#### Stability

Several participants described that the daily visits re-introduced order and stability into their lives, particularly compared to the chaotic lifestyle “on the scene.”

‘To have any stability at all, to have something to do during the day. At least one thing I have to go to. That's actually a good thing for me. So, I could also say that I only come once a week and have the rest as [oral] carries. But no, it’s good when I always come here’ (Julia, in iOAT for one year).

Some felt that the daily routine of visits to the clinic prepared them to re-enter the job market and establish a “normal” life. Moreover, participants often commented that the mere fact of “keeping busy” distracted them from taking street drugs and helped them to stay clean. A small group of participants additionally stated that regular external control measures (e.g., daily alcohol tests conducted prior to DAM-application) facilitated their abstinence. For these participants, changing iOAT's strict regime was undesirable. One participant, for instance, described that ending iOAT

‘… would be deadly. If, if that would stop, now that- then the swigging [alcohol abuse] is right back on again, because I don’t have to go to the doc twice [a day]. […] If I still have to come here with my rollator [wheeled mobility aid] when I’m 70, then I’ll just come here. And jam the rig [needle] in’ (Ralf, in iOAT for 5 years).

#### Social contact

Another aspect that prompted positive comments from participants was the social contact with other patients and the clinics' staff during their visits. They contrasted this with the hectic and solitary life “on the streets” and with negative experiences from former treatment attempts.

‘Sometimes it feels as if that was even more important to me than taking the diamorphine here. That I always have a point during the day where I can talk, even if it’s just for a moment. That really does me good […]. That you just see them here every day and can talk openly. That's also how a lot of topics come up. Or, with me it has happened that they saw that I was doing really bad: “Come on then, go in and have a talk with the doctor in there”. So, they also check up on you so that you don’t slip through somehow’ (Angela, in iOAT for 2 years).

### Negative aspects

#### Inconveniency

Most participants found the daily visits inconvenient. This mainly regarded the need to consider iOAT in planning their daily lives and the time “lost” during commutes or while waiting in line at the clinic.

‘Coming here twice a day, that’s- When you came here the first time and you go home, it’s always like, always breathing down your neck and ruining the day a bit. Just like, when you know “I have to go ag- again”. […] And actually, you only feel calm when you go home after the second time’ (Horst, in iOAT for 1.5 years).

Additionally, some disliked daily encounters with other patients. These participants perceived patients who continue to use and sell street drugs as threatening to their abstinence. Other participants did not have a problem with the daily visits per se but felt burdened, for instance, by train rides during rush hour due to their social anxiety or mobility impairments.

#### iOAT complicating regular employment

A further prominent negative aspect was the perceived incompatibility of repeated visits to the clinic while maintaining a regular job, which was a reason for some participants to consider ending iOAT.

‘I would just like to start doing a job again, probably. Even if- It doesn't have to be full-time again, but maybe just a small job in addition to [social benefits]. But in a way that I just come once a week or twice a week and then have my [oral] take-home. That would just be a goal that I would like to achieve’ (Tobias, in iOAT for 6 years).

When working regularly, participants sometimes described the burden of having to reconcile their work schedule with visits to the clinic. This was more complicated when participants preferred not to disclose their treatment status to employers, e.g., due to fears of stigmatisation and job loss.

‘You have to make sure that you, that the workplace will put up with it. Of course, they can’t know it. But when you come here twice or even just once, you have to make sure that it somehow works out timewise. […] You have to be careful, put your plaster away and stuff like that. Not that someone sees that when you have a plaster like that every day, they will think- What would you think if your colleague had a plaster like that on every day or like me now [showing own needle tracks]. You have to be careful that nobody notices at work’ (Barbara, in iOAT for 2 years).

### iOAT as the best alternative option

Despite these impediments, most participants described missing a dose of DAM as highly unusual or never occurring. They often explained their high adherence and commitment to the treatment regime with a lack of preferrable alternatives. For instance, some participants reversed the argument of time “lost” during clinic visits by considering their practical alternatives.

‘I always say, I have nothing to do anyway. And if I sit at home all the time and watch TV, I can also do this here’ (Markus, in iOAT for 2.5 years).

This perspective was also reported in regard to negative experiences that the participants had before entering iOAT, e.g., during previous treatment attempts and/or the use of street drugs. Past experiences of craving, withdrawal, and illegal activities to obtain drugs rendered the daily visits “unnegotiable”, with participants feeling that iOAT's benefits far outweighed any inconveniences.

‘For me, [entering iOAT] was a step forward and having freedom again, having independence again. Even though it might not sound like that at first, that you have to do something. Every day. And really every day. Really. Many people don't understand that. But it really is the case that you have to go every day and, uh- I think in the end you like going there because you know it spares you from a lot’ (Natalie, in iOAT for 7 years).

### Facilitators of daily visits

Apart from their degree of treatment engagement being driven by an absence of preferable alternatives, participants described several elements that facilitated compliance with iOAT's requirements. These facilitators were specific factors that eased the burden of daily attendance and helped participants to manage the challenges associated with frequent clinic visits.

#### Therapy on a level playing field

A central facilitator of daily visits was what we refer to as "Therapy on a level playing field." This encompasses a range of interconnected factors that create a supportive and respectful treatment environment. In this regard, participants highlighted the importance of the clinic staff's approachability and friendliness. They often described how the clinics' welcoming environment and non-punitive approach played a significant role in easing their discomfort about daily visits and the supervised application.

‘Usually, when I come here, on the way I think "ah, I don't feel like it, I don't want to". But when I'm here, when I leave the lift upstairs, it's like "click", that changes. When there are people there, because [they are] friendly. Like, "hello" and stuff, then it changes somehow. That's why. Because the atmosphere here is really good. So, not coming here, I don't do’ (Julia, in iOAT for 1 year).

Particularly when they had had negative experiences in former treatment facilities, participants valued clinic staff's humane and respectful attitude and being treated as individuals with unique needs and preferences. Contributing to a sense of autonomy, this encouraged participants to customise iOAT to their individual needs and allowed them to better cope with the therapy's requirements. Here, participants frequently mentioned supplementing iOAT with oral substitutes to reduce the frequency of visits, which helped some participants to reconcile iOAT with a regular job.

‘I come in the morning and take a very small dose before work. Like 50 [mg of DAM]. And when I'm not working, I take 130 [mg of DAM] in the morning. And when I'm working, I take a little bit more Polamidon [Levomethadone] instead’ (Benjamin, in iOAT for 4.5 years).

Yet, this was not an option for those participants who rejected oral substitutes altogether, e.g., due to past negative experiences in oOAT. For these participants, flexibility in deciding when and how often to come to the clinic mitigated feelings of being controlled and enhanced their sense of self-efficacy.

‘I just take my dose in the morning and my dose in the evening and then that's enough for the day, that usually works. So, sometimes I don't even do a third dose, sometimes I just do two doses and I vary that depending on my craving’ (Florian, in iOAT for 8 months).

‘With Pola [Levomethadone] and all, going there every day and then only during a certain time, that was more complicated. Now I have more freedom, can come whenever I want’ (Jürgen, in iOAT for 1.5 years).

#### Multidimensional care

Several participants described how their clinics' multidimensional approach was crucial to cope with iOAT's requirements. This reflects the integration of various services at the clinic beyond DAM application to address patients' multifaceted needs. Within the broader context of multidimensional care, a central component mentioned by many participants was the reduction of travel distances and time spent commuting. This was particularly important for those living with physical impairments, e.g., bound to a wheelchair or otherwise living with chronic diseases. By scheduling various appointments in the clinic, daily visits transcended the DAM-application itself and became an efficient way to handle participants' complex physical, psychological, and bureaucratic matters.

‘I have everything here in the house, so I don't have to go anywhere else. I have my drug counsellor here, I have my doctor here, I have- Yes, here I have most of what I need’ (Thomas, in iOAT for 4 years).

Several participants additionally found the option to have breakfast and lunch, possible in the Stuttgart clinic, helpful to “make the best” of visits to the clinic. These participants felt like eating in the treatment facility freed up time otherwise spent buying and preparing food and perceived communal meals as an important part of the supportive community in the clinic. This could be the basis for why some participants described the clinic as the “centre of their lives”.

#### Strategic lifestyle choices

Strategic lifestyle choices related to housing, employment, and treatment location represent a final aspect facilitating participants' ability to integrate frequent clinic visits into their daily lives. When given the chance to choose between treatment facilities, several participants in Berlin (where two iOAT-clinics exist) reported to have selected the clinic in closer proximity to their homeplace. Similarly, participants in Stuttgart and Berlin described how they prioritised flats near their iOAT-clinic when looking for a new home, e.g., when transitioning from assisted to individual living. Likewise, when participants re-entered the job market, they preferred employment near their clinic. Barbara emphasized that she chose her new job.

‘… because it's right next to the [clinic] and I only have to walk over here during my break. That's why it works out pretty well.’

### Suggestions for Improvement

Despite the positive aspects of on-site dosing and the facilitating factors mentioned above, participants' suggestions for improving iOAT often concerned the repeated visits to the clinic. Some suggested expanded opening times to accommodate early working hours or flexibly adapting iOAT's frequency, e.g., taking DAM only once or twice a month depending on one's craving. However, most suggestions regarded take-home arrangements for injectable DAM.

Generally, participants stated that the possibility to inject DAM at home would increase their personal freedom.

‘Then you wouldn't be so bound to this place. Because that's also again a route. To come here when you don't live nearby’ (Ronald, in iOAT for 9 years).

Participants also believed that take-home injectable DAM would enable them to better meet their individual needs. Some stated, for instance, that they would prefer injecting DAM directly before going to sleep or in an environment of their choice. Participants who dislike oral substitutes additionally wished for carries to avoid having to switch to oral medications when being hospitalised, on holidays, or otherwise unable to come to the clinic. Furthermore, participants stated that the possibility to inject DAM outside of a clinical environment would help them to pursue a stable job and, for some, would thus be the deciding factor for (dis-)continuing iOAT.

At the same time, however, several participants voiced concerns over take-home arrangements for injectable DAM and expressed understanding for the current regulations. They felt as though clinical supervision was necessary in case of side effects such as respiratory depression and to prevent the diversion or misuse of DAM. Many participants thus described take-home injectable DAM as desirable, yet unattainable.

‘Of course, it would be great if you could get it as take-home. But I can understand that, because then people shoot [inject] themselves to death at home because they take three days' dosage in one day. They're dead and then this place is closed. I understand that that doesn't work’ (Sebastian, in iOAT for 7 years).

Some participants additionally advised against take-home arrangements due to the positive aspects of daily visits. They were concerned that the absence of daily routines, tasks, and social support would outweigh any benefits of injecting DAM at home.

## Discussion

We found that daily visits in iOAT are not experienced uniformly. Daily visits gave a sense of structure and normalcy for some participants, particularly when compared to previous experiences with unstable OAT and illicit drug use [33, 35, 51, 52]. Additionally, some participants felt as though “keeping busy” and being subjected to external control measures contributed to reaching their goal of abstinence [34]. Finally, participants valued that daily visits provided access to social support and long-term, comprehensive care [36, 39].

However, as described in previous studies [24, 25], most participants felt impeded in their daily lives by receiving DAM on-site only. Our findings contextualise previous quantitative studies on iOAT and shed light on, for instance, the considerable effort required of patients to achieve quantitative improvements in “social integration” in iOAT [53]. Resonating with Dennis [16], participants were often forced to make profound lifestyle choices (e.g., on where to live or work) and structure their lives around iOAT in order to cope with the treatment regime. This was complicated by the limited number of iOAT clinics in Germany [6], which restricted the potential to make empowering choices as to where to receive treatment [54]. A further aggravating factor was the intersecting sources of stigma patients faced, which was particularly apparent for those trying to reconcile iOAT and formal employment [55, 56]. Our findings thus resonate with previous literature on interconnections between OAT policies and structural stigma [57, 58]. These factors should be acknowledged in all considerations related to iOAT dosing regulations.

Participants' general acceptance of the impediments accompanying iOAT is notable, as daily visits in iOAT are not mitigated by the possibility of contingent take-home doses [44, 45]. This acceptance might be due to iOAT-patients' relative lack of alternative options: participants often reflected upon iOAT and the rules it imposes relative to past negative experiences with oral substitutes, street heroin use, or withdrawal. Largely, they concluded that now, their life was undoubtedly better than before. Compliance due to a lack of alternative options has already been described for oOAT [35, 59, 60] and iOAT [61]. This is likely to be increased in iOAT considering its image as a therapy of “last resort” [62]. However, being the “least bad option” should not be the main driver for treatment engagement [63]. Below, we thus explore how to adapt iOAT to render it more responsive to patients' needs.

We found that the clinics' approach could mitigate the practical negative impacts of daily visits and the feeling of being controlled or punished [19, 24, 33]. Here, participants highly valued collaboration with healthcare staff to help customise their medication. This often involved combining iOAT with oral substitutes, which has previously been described as a strategy to reduce clinic visits in iOAT [8, 61]. When oral substitutes were not an option for participants, flexible and extended opening times were crucial to reconcile iOAT with individuals' goals and responsibilities [40, 41]. Our findings underline the importance of providing non-judgmental and flexible OUD-care “on a level playing field” that encourages self-determination [64–66]. Furthermore, by providing iOAT as a complex intervention to address mental and physical health in addition to legal and social aspects [67], treatment engagement was facilitated [33]. Receiving multidimensional care in one place reduced commutes, which was of particular importance for individuals living with mobility impairments [43]. Providing holistic care in-house to make clinic visits more “efficient” [38, 42] and to ease the “work” necessary to self-manage chronic diseases [68] are factors likely to become even more relevant as patients in iOAT become older with increasing co-morbidities [69, 70]. However, in light of current legislative frameworks, there are limits to how clinics and their staff can support clients in iOAT [66, 71]. Furthermore, a balance must be struck between providing comprehensive care within clinics and ensuring that patients are integrated into their wider communities. Despite social bonds formed within clinics, efforts to facilitate daily visits and make iOAT-clinics the “centre of one's life” might simultaneously impede patients' community engagement [38] and contribute to the social segregation of those living with OUD [29].

Underscored by participants' wishes for more flexible dosing policies, it is thus worthwhile to consider alternatives to the current approach towards iOAT-delivery. One possibility could be unsupervised take-home injectable DAM. However, as emphasized by several participants in this study and by previous research [2], this might be overly risky. Firstly, injected substances in general [72], and DAM in particular [14, 73], bear a higher risk of overdoses. Under certain circumstances (e.g., fever, interaction with other substances, sleep deficit, or prescription errors), unsupervised injectable DAM might therefore threaten patients' safety. Moreover, although unsupervised iOAT has been possible in the UK in the past, this is increasingly being replaced by supervised-only iOAT [74, 75]. Secondly, the possibility of misuse and diversion must be acknowledged. For other substitutes, problems related to diversion are poorly understood [21, 76] and are claimed to be exaggerated [77, 78], which sometimes leads to inappropriate policies [79, 80]. However, due to differing safety profiles and forms of application, it remains questionable if this is transferable to injectable DAM. For instance, the problem of blood borne diseases spreading through misused OAT medications [13] is likely amplified in the context of iOAT. Accordingly, as discussed in prior research [12], participants' concerns that the detriments of take-home injectable DAM might threaten other patients, their clinics' existence, and their own future treatment should not be taken lightly.

Nevertheless, flexible dosing policies in iOAT must not be restricted to current all-or nothing debates [16, 17]. To advance patient-centred iOAT [81] and accommodate individual needs with adequate safety measures, it is helpful to consider approaches from other OAT modalities. Telemedicine-based oOAT—including remote urine drug screening [82]—has been piloted and implemented in the past, particularly in response to the COVID-19 pandemic [83–85]. Despite greater challenges for implementation compared to oOAT, for selected patients remote supervision in iOAT might enhance convenience while maintaining safety, stability, and a close therapeutic alliance. Furthermore, it might be worthwhile to consider DAM-delivery services [86–88] for individuals with mobility restrictions, particularly during in-patient treatment, isolation, or hospice care [89].

To date, there is little evidence on alternative approaches to iOAT [17, 36] and further research should evaluate its acceptability among patients, clinicians, and policymakers [9, 90]. Considering the immense “treatment burden” [22] currently experienced by individuals receiving iOAT, we strongly recommend a comprehensive examination of alternative modes for delivering iOAT. Flexibly meeting patients' individual needs through a variety of dosing models and the balanced expansion of facilitating measures in iOAT-clinics would undoubtedly improve the conditions of iOAT for recipients and increase its appeal for those not yet engaged in treatment.

## Strengths and limitations

A major contribution of our research which can inform clinical and political decision making is the contextualisation of past quantitative studies on iOAT. Our exploratory approach reduced bias by allowing us to investigate facets of iOAT that we had not anticipated. By asking open questions and allowing participants to set priorities during the semi-structured interviews themselves, we aimed to capture what was important for participants at the time of the interview. Nevertheless, this also meant that participants were neither systematically nor exhaustively asked about their thoughts on supervision, on-site application, and frequent visits to the clinic. Consequently, further relevant aspects may have been overlooked and we invite future studies to enrich our findings. We anticipate that ethnographic methods will be particularly enlightening to explore the practical effect of different dosing policies. As for any qualitative study, our findings are geographically and temporally situated and should be cautiously transferred to settings beyond our study sites. Nevertheless, the considerable overlap between both study sites and with research from other contexts suggests that our results are transferable at least to some extent.

By employing a random sampling approach, we aimed to interview participants with a wide spectrum of experiences, including those who might have been hesitant to self-select for engaging in research. There are, however, some limitations inherent to our strategy of participant recruitment. While we emphasised that participation was entirely voluntary and unrelated to participants' care, we acknowledge that the involvement of clinic staff may have influenced the decision of some individuals to participate. Our research is in line with the aspiration of participatory health research to support positive sociopolitical changes for those we focused on in our study [91]. Even so, the participation of people with lived experiences was limited to a “consult” level [47]. While inclusion, for instance by involving peer researchers into our study, would have been favourable, it was not realised due to concerns of confidentiality and the limited resources available for this project.

All self-reported data can be influenced by both interviewer and participant bias. ZF was not involved in the participants' care, avoided leading questions, and stressed that she would not share identifiable data with clinic staff. Nevertheless, social desirability responses during the interviews and consultation of people living with OUD might have influenced what we found, particularly regarding comments on interpersonal aspects, the clinics' organisational structure, or individual goals. In addition, as participants who had discontinued iOAT were under-represented compared to those stably engaged in iOAT, our results are likely biased towards people who can sufficiently cope with and/or profit from the requirement of on-site application in iOAT. Although there was substantial consistency between comments from participants currently in iOAT and participants who discontinued iOAT, the practical significance of daily visits for those currently not or only unstably engaged in treatment can only be addressed here in a limited sense. Notwithstanding, we believe that our aim, to provide rich insights into participants' lived experiences of dosing policies in iOAT, has been achieved.

## Conclusions

We explored the real-life significance of current dosing policies for patients in iOAT. Experiences of on-site application were dynamic and context-dependent, and participants in this study were willing to make far-reaching adjustments to their lives to meet iOAT's requirements. Although on-site application in iOAT has some advantages, it concurrently impedes patients' self-determination and quality of life. Undoubtedly, the management of iOAT is a complex issue for policymakers, clinicians, and patients alike, and reaching a balance between regulation and individualisation is of paramount importance. Nevertheless, current policies might hinder treatment engagement for a relevant proportion of individuals who could greatly benefit from iOAT. More flexible and context-sensitive regulations should be considered and carefully evaluated to free iOAT from being the “least bad option” and make it a therapy that patients truly embrace during their often long-lasting treatment journey.

### Supplementary Information

**Additional file 1:** Full interview guides.

## Data Availability

The datasets generated and/or analysed during the current study are not publicly available to protect study participants' identity. Anonymised data can be made available from the corresponding author on reasonable request.

## Abbreviations

DAM: Diamorphine
OAT: Opioid agonist treatment
iOAT: Injectable opioid agonist treatment
oOAT: Oral opioid agonist treatment
OUD: Opioid use disorder
