# Supplementary material for: Supervised on-site dosing in injectable opioid agonist treatment-considering the patient perspective. Findings from a cross-sectional interview study in two German cities
Source: Harm Reduct J. 2023 Nov 1;20:162. doi: 10.1186/s12954-023-00896-6 (PMC10619267; doi:10.1186/s12954-023-00896-6)
Supplement: Supplementary file 1 — Additional file 1: Full interview guides. [file 12954_2023_896_MOESM1_ESM.docx]

Supplementary Material 1: Full interview guides

**For participants currently in SIOT**

What was your life like when you initiated SIOT?

Why did you decide to initiate SIOT?

What were your expectations, what were your goals?

Was there anything you worried about?

What is your experience with SIOT?

Do you think that it has helped you in any way?

If so, how? What has helped you the most?

If not, why not?

What do you like/dislike? What problems have you experienced?

How do you think SIOT could be improved?

Is there any additional support you would like to get but you are not receiving currently?

Did you ever think about ending the treatment?

If so, why? Can you tell me a bit more about that?

Is there anything else you would like to comment on that I have not asked you about?

**For participants currently in oral opioid substitution treatment who received SIOT in the past**

What was your life like when you initiated SIOT?

Why did you decide to initiate SIOT?

What were your expectations, what were your goals?

Was there anything you worried about?

What was your experience with SIOT?

Do you think that it has helped you in any way?

If so, how?

If not, why not?

What did you like/dislike? What problems have you experienced?

How do you think SIOT could be improved? What do you think would have made your experience in SIOT better?

Can you tell me a bit more about the time you ended the treatment?

Would you consider going back into treatment?

If so, why?

If not, why not?

Is there anything else you would like to comment on that I have not asked you about?

**For participants currently in oral treatment who have never been in SIOT**

What was your life like when you initiated substitution treatment?

Why did you decide to initiate substitution treatment?

Are you aware of SIOT?

If so, what do you think of it?

Would you like to receive SIOT?

If so, why?

If not, why not?

Is there anything that would need to change for you to consider starting SIOT?

Is there anything else you would like to comment on that I have not asked you about?
